# Dynamic miRNA profile of host T cells during early hepatic stages of *Schistosoma japonicum* infection

**DOI:** 10.3389/fimmu.2022.911139

**Published:** 2022-09-02

**Authors:** Bikash R. Giri, Shun Li, Chuantao Fang, Lin Qiu, Shi Yan, Maria Y. Pakharukova, Guofeng Cheng

**Affiliations:** ^1^ Shanghai Tenth People’s Hospital, Institute for Infectious Diseases and Vaccine Development, Tongji University School of Medicine, Shanghai, China; ^2^ Key Laboratory of Animal Parasitology of Ministry of Agriculture and Rural Affairs, Shanghai Veterinary Research Institute, Chinese Academy of Agricultural Sciences, Shanghai, China; ^3^ Shanghai Institute of Nutrition and Health, Chinese Academy of Sciences, Shanghai, China; ^4^ Institut für Parasitologie, Veterinärmedizinische Universität, Wien, Austria; ^5^ Institute of Cytology and Genetics, Siberian Branch of Russian Academy of Sciences, Novosibirsk, Russia; ^6^ Department of Natural Sciences, Novosibirsk State University, Novosibirsk, Russia; ^7^ Institute of Molecular Biology and Biophysics, Novosibirsk, Russia

**Keywords:** *Schistosoma japonicum*, T cell, infection, immune response, microRNA

## Abstract

Schistosomes undergo complicated migration in final hosts during infection, associated with differential immune responses. It has been shown that CD4^+^ T cells play critical roles in response to *Schistosoma* infections and accumulated documents have indicated that miRNAs tightly regulate T cell activity. However, miRNA profiles in host T cells associated with *Schistosoma* infection remain poorly characterized. Therefore, we undertook the study and systematically characterized T cell miRNA profiles from the livers and blood of *S. japonicum* infected C57BL/6J mice at 14- and 21-days post-infection. We observed 508 and 504 miRNAs, in which 264 miRNAs were co-detected in T cells isolated from blood and livers, respectively. The comparative analysis of T cell miRNAs from uninfected and infected C57BL/6J mice blood showed that *miR-486b-5p/3p* expression was significantly downregulated and linked to various T cell immune responses and *miR-375-5p* was highly upregulated, associated with Wnt signaling and pluripotency, Delta notch signaling pathways, *etc.* Whereas hepatic T cells showed *miR-466b-3p*, *miR-486b-3p*, *miR-1969*, and *miR-375* were differentially expressed compared to the uninfected control. The different expressions of some miRNAs were further corroborated in isolated T cells from mice and *in vitro* cultured EL-4 cells treated with *S. japonicum* worm antigens by RT-qPCR and similar results were found. In addition, bioinformatics analysis combined with RT-qPCR validation of selected targets associated with the immune system and parasite-caused infectious disease showed a significant increase in the expression of *Ctla4*, *Atg5*, *Hgf*, *Vcl* and *Arpc4* and a decreased expression of *Fermt3*, *Pik3r1*, *Myd88*, *Nfkbie*, *Ppp1r12a*, *Ppp3r1*, *Nfyb*, *Atg12*, *Ube2n*, *Tyrobp*, *Cxcr4* and *Tollip*. Overall, these results unveil the comprehensive repertoire of T cell miRNAs during *S. japonicum* infection, suggesting that the circulatory (blood) and liver systems have distinct miRNAs landscapes that may be important for regulating T cell immune response. Altogether, our findings indicated a dynamic expression pattern of T cell miRNAs during the hepatic stages of *S. japonicum* infection.

## Introduction

*Schistosoma* has a complex life cycle and needs different hosts (intermediate host and definitive host) to complete its life cycle. It has fascinating migratory nature starting from the infection site of the skin epidermis to blood vessels, then through the heart and lungs to the vasculature of the livers (1). During the migratory process, significant morphological changes and worm developments are associated with parasites (2). Ultimately, the parasites can develop into adult worms and then survive within the venous system of the definitive mammalian hosts for many years (3). The circulatory system is accumulated by various immune defenses, including immune cells, phagocytes, complement proteins, and antibodies. The adult schistosomes are shown to adopt several strategies, from coating their outer tegument with antigens from the hosts to secreting excretory-secretory products and extracellular vesicles to modulate the host immune response in its favor (4).

Livers are known as immune-permissive organs with unique anatomy, which contain various immunocompetent cells such as dendritic cells, Kupffer cells (KCs), natural killer (NK) cells, natural killer T cells, regulatory T cells, *etc*. (5, 6). Schistosomes have complicated migration in final hosts. Upon cercaria penetrating the skin epidermis, juvenile schistosomula are transformed and then reaches the dermal blood vessels. Then schistosomula reach the lungs and *S. japonicum* schistosomula found in lungs at day 2 and peaks at 3 days post-infection. Subsequently, S. *japonicum* schistosomula usually migrate into the liver 3 days post-infection and take 8-10 days for them to grow up and develop in the livers. Then, parasites begin to lodge in the portal and mesenteric veins at 11 days post infection. The early phase of *S. japonicum* living in the hepatic portal vein is essential to find a mate and pairing and complete maturation as well as flow to reach the egg laying sites (2, 7). The majority of studies focused on *S. japonicum* eggs induced immune response and liver pathology in final hosts (8), however, early stages of hepatic progression and related host immune response, which is critical for parasite development and maturation, remains poorly characterization. Consequently, it is necessary to determine how *S. japonicum* infection induces host immune response at early hepatic schistosomula stages, which may help to further reveal the relationship between schistosomula modulating host immune response and parasite development.

T helper cells have a crucial role in shaping the immune responses during schistosomiasis (9). During the early phase of infection, cercaria initiates a Th1 immune response, characterized by increased pro-inflammatory cytokines, including TNF-α and IFN-γ, IL-1, and IL-6 (10). When worms develop into adults and lay eggs, however, the Th2 immune response is triggered by their soluble egg antigen (11, 12). The Th2 immune response plays a critical role in the pathogenesis of schistosomiasis (13). Previous studies have suggested that Th17/IL-17 exacerbates egg-induced liver pathology and treatment with anti-IL-17 antibodies remarkably inhibits hepatic granulomatous inflammation (14). Then, Treg cells are recruited in the liver to hepatic granulomas and exert an immunosuppressive role to limit granulomatous inflammation and fibrosis (15, 16). Moreover, recent studies showed that Tfh and Th9 cells potentially promote liver granulomas and fibrogenesis in *S. japonicum* infected murine model of schistosomiasis (17, 18). Overall, these studies suggested that T cell subsets undergo complex crosstalk with antigen-presenting cells that regulate the pathological progression of schistosomiasis (19).

MicroRNAs (miRNAs) are endogenous small non-coding RNAs that regulate various biological processes, including proliferation, development, differentiation, and cell death, *etc*. (20). In the murine liver, some miRNAs such as *miR-146b* and *miR-155* are dysregulated during the mid-phase of schistosome infection, indicating they are potentially involved in the modulation of hepatic inflammation (21, 22). Additionally, some studies have suggested that specific miRNAs can regulate T-cell activation, proliferation, and development by targeting prime transcription factors, signaling molecules, and cytokines (23, 24). For instance, the ablation of mature miRNAs at the early thymocyte developmental stage leads to the developmental arrest and a consequent peripheral mature alpha-beta T and invariant natural killer T (iNKT) cell pool (25–28). Furthermore, the enhanced expressions of *miR-146b* and *miR-155* may induce the recruitment of lymphocytes (B and T lymphocytes) in response to antigens secreted by eggs (29, 30). Besides, the miRNA expression profile of thymic T cells at each developmental stage shows a unique pattern of expression (31). Overall, these studies suggested that miRNAs can regulate T cells differentiation and functions. However, the detailed repertoire of T cell miRNAs has not been explored yet during *Schistosoma* infection particularly for early stage. Understanding of mechanisms of miRNAs mediated T cell immune response during *S. japonicum* infection may help to develop effective strategies against schistosomiasis.

Here, we reported the comprehensive repertoire of T cell miRNA profiles from blood and livers of C57BL/6J mice during *S. japonicum* infection at 14 dpi and 21 dpi using fluorescence-activated cell sorting (FACs) combined with deep sequencing. In comparison to uninfected control, we identified several miRNAs that are differentially and enriched explicitly in T cells of *S. japonicum*-infected mice. The results indicated the dynamic expression profiles of T cell miRNAs in blood and livers, exhibiting unique regulatory signatures during *S. japonicum* infection at early hepatic schistosomula stages (14 dpi and 21 dpi).

## Materials and methods

### Establishment of schistosomiasis mice model

Male C57BL/6J mice (6–8 weeks old) were procured from Shanghai SLAC Laboratory of Animal Co., Ltd, (Shanghai, China). All animals were housed under standard experimental conditions. All animal experiment protocols were approved by the Animal Management Committee and the Animal Care and Use Committee of the Shanghai Science and Technology Commission of the Shanghai Municipal government for Shanghai Veterinary Research Institute, Chinese Academy of Agriculture Sciences, China (Permit No. SHVRI-SZ-20200720-03). The life cycle of *S. japonicum* (Anhui isolate) was maintained in male mice and the intermediate snail host *Oncomelania hupensis* (Center of National Institute of Parasitic Disease, Chinese Center for Disease Control and Prevention, Shanghai, China). The mice were challenged with 50 ± 2 *S. japonicum* cercariae *via* abdominal skin.

### Purification of T cells from peripheral blood and liver

A total of two biological replicates were used for each group (the sample for each biological replicate is pooled from 10 mice, n = 10). At 14- and 21-days post-infection (dpi), blood samples were collected from *S. japonicum* infected mice in anticoagulant blood collection tubes (BD Biosciences, Mountain View, CA, USA). Similarly, blood samples of uninfected mice were collected as a control. The whole blood was diluted with PBS and overlaid on top of the Ficoll (1.084). Then centrifuge at 400 × *g* for 30-40 min at room temperature, during the centrifugation, granulocytes, platelets and red blood cell (RBC) pellet to the bottom of the tube and the peripheral blood mononuclear cells (PBMCs) float over the Ficoll-plasma interface layer. After collection, PBMCs were washed with PBS at 300 × *g* at 4°C twice. Then PBMCs were lysed using RBC lysis buffer (Biolegend, San Diego, USA) and the remaining cells were pelleted and resuspended in 200 μL of wash solution. The fluorochrome-conjugated antibodies against mouse (BV510, Fixable Viability Stain 510, BD Biosciences, Mountain View, USA), CD45, CD45R (B220), CD3e, CD4 and CD8a (eBioscience™, Frankfurt, Germany) with the dilutions as suggested by manufacturer was used to stain immune cells and then cells were sorted using a BD FACsAria II system (BD Biosciences, Mountain View, USA). Firstly, we sorted the live cells by gating BV510 positive cells and then interrogated for surface CD45 expression to sort leukocytes. Later T and B cells were sorted using CD3e and B220 antibodies. Then the CD3e positive population was gated using CD4 and CD8a antibodies to sort T cells. The flow cytometry data were analysed using FlowJo (v10.6.2).

For isolating liver T cells, both infected and uninfected mice were anesthetized, and livers were perfused with RPMI-1640 (Invitrogen, USA) at indicated days of post infection (dpi). The livers were thoroughly washed, minced into small pieces with surgical scissors, and forced through a 70 µm cell strainer (Falcon) using a sterile syringe plunger. The obtained preparation was suspended in 50 mL RPMI-1640 medium and centrifuged for 5 min at 700 *× g* at 4°C. Then, the pellet was resuspended with 30 mL of 40% Percoll (GE Healthcare, Boston, USA), recentrifuged for 20 min at 900 × *g* at 4°C with the off-brake setting twice. The resultant sediments were resuspended in RPMI-1640 and filtered through a 40 µm cell strainer (Falcon). The pellet was resuspended again in 2 mL of RBC lysis buffer (Biolegend, San Diego, USA), incubated for 5 min, then centrifuged for 5 min at 500 *× g* at 4°C. Finally, the pellet obtained was resuspended in 1 mL PBS and the antibodies used in above sections were added with same dilutions and incubated for 30 min at 4°C. After staining, cells were washed with PBS containing 0.04% BSA at 500 *× g* for 5 min at 4°C. The collected cells were resuspended and sorted by flow cytometry as described for T cell purification. The information of antibodies used are listed and provided in **Supplementary Table 1**.

### RNA preparation, sequencing and data analysis

Total RNAs were extracted from sorted peripheral blood T cells and liver T cells (14 dpi, 21 dpi and uninfected control) using a miRNeasy Mini Kit (QIAGEN, Germany). RNA quality was evaluated using an Agilent 2100 Bioanalyzer (Agilent Technologies). Small RNAs in the 18-30 nt fraction were extracted from denaturing 15% polyacrylamide gels and used for library preparation using the TruSeq^®^ Small RNA Library Preparation Kit (Illumina, CA, USA). Twelve resulting small-RNA libraries were subjected to Illumina 50 bp single-end sequencing by Illumina HiSeq™ 2500 sequencing at BGI (The Beijing Genomics Institute). Following sequencing, raw reads were cleaned by removing adapter sequences, reads containing poly-N, low-quality reads, and oligonucleotides with length >32 or <18 nt. The remaining sequences were mapped to the reference genome and another small RNA database, including miRbase, siRNA, piRNA, and snoRNA with Bowtie (32). The covariance models (cm) search was mainly performed for Rfam mapping (33). The software miRDeep2 (34) was used to predict novel miRNA by exploring the secondary structure. The small RNA expression level is calculated by counting absolute numbers of molecules using unique molecular identifiers (35). Differential expression analysis was performed using the DEGseq (36), Q value ≤ 0.05, and the absolute value of Log2Ratio ≥ 1 as the default threshold to judge the significance of expression difference. RNAhybrid (37), miRanda (38) and TargetScan (39) were used to predict the target genes of miRNAs. To annotate gene functions, the target genes were aligned against the Kyoto Encyclopedia of Genes (KEGG) and Gene Ontology (GO) database (40, 41). GO enrichment analysis and KEGG enrichment analysis of target genes were performed using phyper, a function of R. The *P*-value was corrected using the Bonferroni method (42), and a corrected *P*-value ≤ 0.05 was taken as a threshold. GO terms or KEGG terms fulfilling this condition were significantly enriched terms.

### Preparation of *S. japonicum* soluble antigen and stimulation of isolated T cells and EL-4 cells

Freshly perfused *S. japonicum* were thoroughly washed in PBS (pH 7.4). PBS solution containing protease and phosphatase inhibitor (Thermo Fisher Scientific Corp., MA, USA) was added and the mixture was homogenized for 30 min on ice and the homogenate was sonicated for 30 min. Then centrifuged at 16,000 x *g* for 30 min and the supernatant was used for *S. japonicum* soluble worm antigen (SWA). Protein concentration was measured by standard Bradford protein assay (Beyotime Biotechnology, China) using bovine serum albumin as standard. T cells were isolated from blood and liver of C57BL/6J mice using CD90.2^+^ MicroBeads (Miltenyi Biotec, Bergisch Gladbach, Germany) following manufacturers protocol. Isolated cells were cultured RPMI 1640 medium containing 10% fetal bovine serum and 1% penicillin-streptomycin solution, Then, the cells were treated with *S. japonicum* soluble antigen (15 µg/mL) or PBS (control) for 36 h and total RNA was isolated and RT-qPCR was performed to assess the miRNA expression. Similarly, EL-4 cells were treated with *S. japonicum* soluble antigen or PBS (control) and RT-qPCR was carried out to assess selected miRNA expression.

### MiRNA and its target gene expression validation by RT-qPCR

A real-time quantitative reverse transcription-polymerase chain reaction (RT-qPCR) was performed to confirm miRNA expressions and the target gene expressions in isolated T cells. Briefly, total RNA was extracted using Trizol (Thermo Fisher Scientific) and reversed transcribed using the miScript II RT Kit (QIAGEN, Hilden, Germany). The miRNA expression was determined using the reverse primer given in the miScript SYBR Green PCR Kit (QIAGEN). For the expressions of target genes, the real-time PCR was performed using the following thermal cycling program: 95°C for 5 min, 40 cycles at 95°C for 10 s, 57°C for 20 s, and 72°C for 36 s. *U6* (a type of small nuclear RNA) and *glyceraldehyde-3-phosphate dehydrogenase* (*GAPDH*) were used as the internal controls. The fold change was calculated using the 2^−ΔCT^ method (43). All the primer sequences are provided in **Supplementary Tables 2**, **3**.

### MiRNA target pathway network building

To build a murine miRNA target network, we used the online tool miRTargetLink 2.0 (https://www.ccb.uni-saarland.de/mirtargetlink2) to build the miRNA target network (44). The strongly validated targets with pathways were included to build and visualize the network of several most significantly differentially expressed miRNAs such as *miR-486b-5p*, *miR-375-5p*, *miR-1969*, *miR-486b-3p*, *etc.*

### Statistical analyses

The RT-qPCR results were expressed as mean ± SEM from representative triplicate experiments. The comparison among uninfected vs 14 dpi and 21 dpi were analyzed using one-way ANOVA and comparative analysis between two groups were analyzed using Student’s T-test. The results at *P* ≤ 0.05 were considered statistically significant.

## Results

### Experimental design and data output

**Figure 1:**
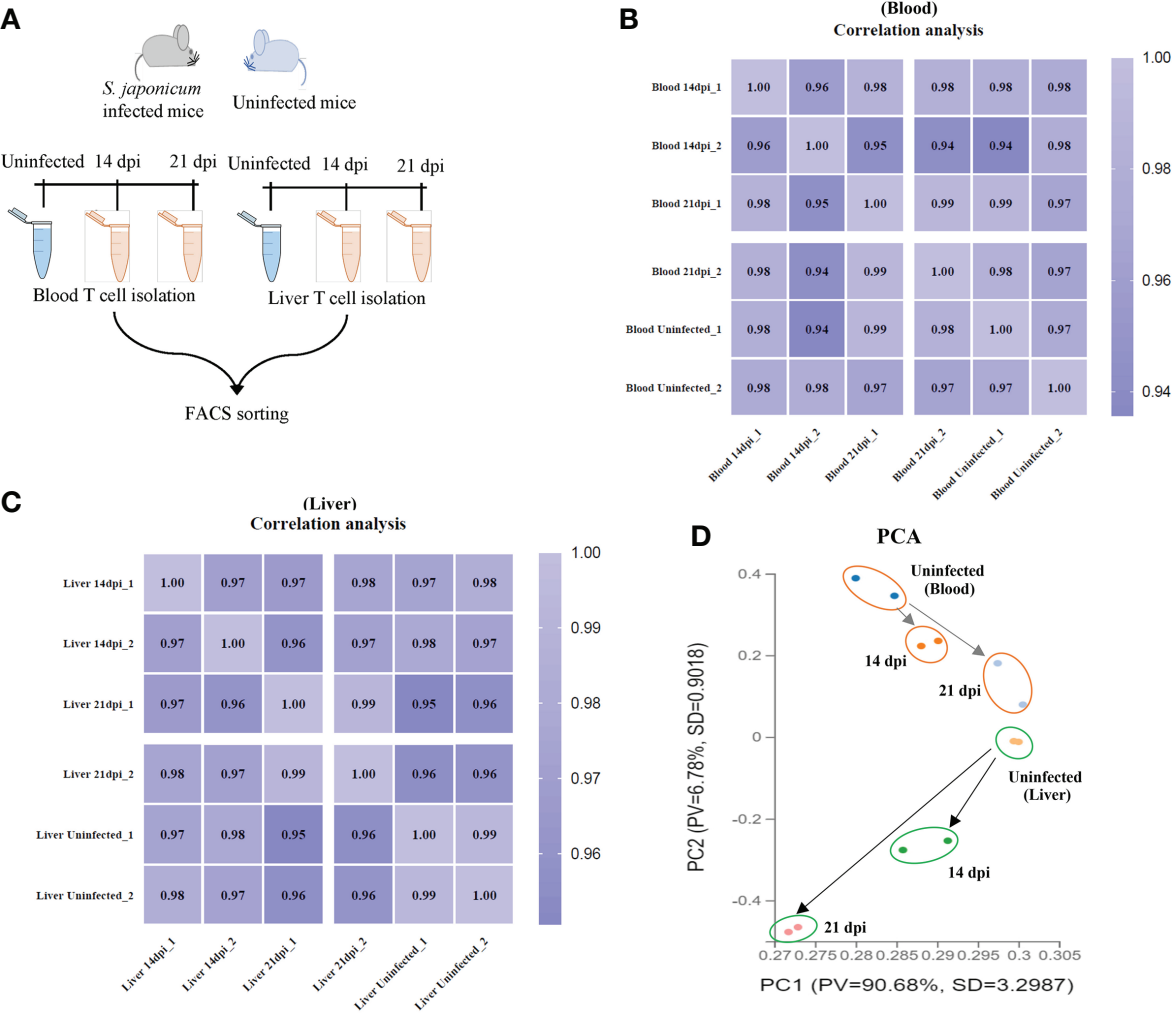
T cell isolation and RNA seq analyses. **(A)** Schematic workflow for T cell isolation at different stages of *S. japonicum* infected mice and uninfected mice; **(B, C)** Heatmap of Pearson correlations of T cell miRNA expressions among different samples from blood **(B)** and liver **(C)** at different stages of *S. japonicum* infected mice and uninfected mice. Colors in the heat map indicate the Pearson correlation coefficient among different samples, lighter color indicated higher correlation; **(D)** PCA analyses of T cell samples isolated from blood and liver of *S. japonicum* infected mice at 14 dpi and 21 dpi and uninfected mice.

T cells were isolated from murine peripheral blood and liver tissues using flow cytometry (the details of workflow and T cell sorting were shown in **Figure 1A** and **Supplementary Figures 1A, B**). The average of live T cells isolated from murine blood were 85.2%, 99.4% and 97.4% at 14 dpi, 21 dpi and uninfected mice, respectively. Similarly, there were 94.4%, 99.6% and 93.95% of average live T cells isolated from livers at 14 dpi, 21 dpi and uninfected mice, respectively (**Supplementary Figures 1C, D**). In addition, we observed the average of live CD4^+^ T cells (66.15%, 50.3% and 66.15% for blood; 74.1%, 60.9% and 57.95% for liver) and CD8a^+^ T cells (25.7%, 22.75% and 26.3% for blood; 10.15%, 20.25% and 15.8% for liver) at 14 dpi, 21 dpi and uninfected mice (**Supplementary Figures 1E** **, F**). Then, the isolated T cells were subject to RNA isolation for RNA-Seq analyses. Details output data of RNA-seq for each library given in **Supplementary Data Sheets 1**, **2**. The average number of obtained clean reads for T cells isolated from blood were approximately 25.92, 29.51 and 28.12 million and were approximately 33.50, 30.95 and 30.78 million for T cells isolated from liver at 14 dpi, 21 dpi and uninfected mice, respectively (**Supplementary Data Sheet 1**). The average percentages of the total mapping clean reads were 87.315%, 91.21% and 92.98% for blood T cells at 14 dpi, 21 dpi and uninfected mice and were 86.465%, 85.18% and 89.28% for liver T cells in corresponding groups, respectively (**Supplementary Data Sheet 2**). The classifications and distributions of small RNAs for each sample given in **Supplementary Figures 2A, B**. The results showed that most reads are related to intergenic regions followed by unmapped, hairpin, mature, rRNA, tRNA and precursor and others. To determine the cluster of miRNA expression profiles among different samples (blood and liver) for different groups (14 dpi, 21 dpi and uninfected mice), Pearson correlation analysis was performed. The correlation heat map showed that infected and uninfected had distinct clusters based on the miRNA expression profiles (**Figures 1B, C**). All samples were subjected to principal component analysis (PCA) to assess variations. The results indicated that each T cell sample isolated from different stages (14 dpi, 21 dpi and uninfected control) or organs (blood and liver) is close together, in contrast, a higher dispersion of samples between blood and liver at different stages or organs was observed (**Figure 1D**).

### MiRNA profiles of T cells from blood of *S. japonicum* infected mice

**Figure 2:**
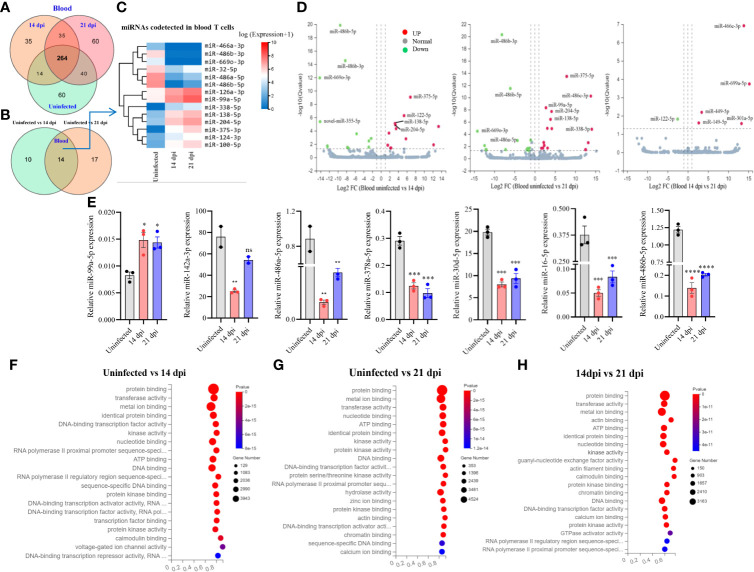
Blood T cell miRNA profiles, expression validation and prediction of molecular functions of their targets. **(A)** Venn diagram showing total and co-detected blood T cell miRNAs among uninfected, 14 dpi and 21 dpi groups (the number indicates the common or specific miRNAs identified in different groups); **(B)** Venn diagram showing codetected and specific blood T cell miRNAs between two groups (uninfected vs 14 dpi and uninfected vs 21 dpi); **(C)** Heatmap showing differentially expressed blood T cell miRNAs co-detected between two groups (uninfected vs. 14 dpi and uninfected vs. 21 dpi); **(D)** Volcano plot showing differentially expressed blood T cell miRNAs for different comparative analyses (uninfected control vs 14 dpi; uninfected control vs 21 dpi; 14 dpi vs 21 dpi). Green dot indicates downregulated miRNAs, red dot indicates upregulated miRNAs and grey dot indicates no significantly expressed miRNAs; **(E)** Validation of increased and decreased expressions of miRNAs of blood T cells by RT-qPCR. Data illustrate representative results and show the mean and standard error mean from an experiment carried out in triplicate. Statistical analysis was performed comparing uninfected vs 14 dpi or 21 dpi using one-way ANOVA and * denotes *P* ≤ 0.05, ** denotes *P* ≤ 0.01, *** denotes *P* ≤ 0.001, **** denotes *P* ≤ 0.0001 and ns denotes non-significant; **(F–H)** GO enrichment analyses of molecular functions of targets for differently expressed miRNAs (Uninfected vs 14 dpi **(F)**; Uninfected vs 21 dpi **(G)**; 14 dpi vs 21 dpi **(H)** in blood T cells). The results showing 20 most significantly enrichment functions of targets for differentially expressed miRNAs.

A total of 508 miRNAs was detected in T cells isolated from blood in *S. japonicum* infected at 14 dpi, 21 dpi and uninfected mice, respectively (**Figure 2A**). Meanwhile, we observed 264 co-detected miRNAs, out of which 50 miRNAs exhibited increased expressions and 81 miRNAs showed decreased expressions compared to the T cells from uninfected control (**Supplementary Figures 2C, D**). In detail, 60 miRNAs were specifically detected in T cells from uninfected control and 21 dpi, whereas 35 miRNAs were specifically detected from 14 dpi (**Figure 2A**). The differentially expressed miRNAs between two groups (uninfected vs 14 dpi: 12 decreased and 12 increased; uninfected vs 21 dpi: 15 decreased and 16 increased) showed the co-detected and specific miRNAs (**Figure 2B**; **Supplementary Datasheet 3**), among them, we noted 14 co-detected miRNAs (mark in green) and two novel miRNAs (*novel-miR-365-5p* and *novel-miR-243-3p*, mark in blue) at 14 and 21 dpi (**Supplementary Figures 3A, B**). The heatmap of co-detected miRNAs revealed 14 differentially expressed miRNAs (7 increased and 7 decreased) in blood T cells among 14 and 21 dpi (**Figure 2C**). Volcano plot analysis indicated that the expressions of *miR-486b-5p/3p* and *miR-669o-3p* were decreased in blood T cells of mice infected with *S. japonicum* (14 dpi and 21 dpi), and in contrast, *miR-375-5p*, *miR-138-5p*, and *miR-204-5p* were upregulated (**Figure 2D**). We further wanted to know whether there are any specific miRNA expression profiles between 14 dpi and 21 dpi, and the results showed significant downregulation of *miR-122-5p*, whereas upregulation of *miR-669a-5p*, *miR-449a-5p*, *miR-301a-5p*, *miR-149-5p*, and *miR-466c-3p* (**Figure 2D**). These results indicated that T cell miRNAs altered their expressions at blood of mice during different stages of *S. japonicum* infection.

Subsequently, we validated the expressions of several miRNAs using RT-qPCR and found that the majority of selected miRNAs (75%) showed consistent expressions with RNA-seq results. As shown in **Figure 2E**, we observed that *miR-99a-5p* expression was significantly increased in T cells of blood from 14 dpi and 21 dpi infected mice compared with uninfected control, whereas *miR-142a-3p*, *miR-486a-5p*, *miR-486b-5p*, *miR-378a-5p*, *miR-16-5p*, and *miR-30d-5p* were decreased. These results were consistent with RNA-Seq data. In addition, analysis of the isolated blood T cells treated with SWA indicated the decreased expressions of *miR-181c-5p, miR-29a-3p, miR-16-5p, miR-30d-5p, miR-142a-3p, miR-151-5p, miR-378a-5p and miR-486b-5p* as compared with that of control **(** **Supplementary Figure 4A** **).** Similar results were also noted in EL-4 T cell treated with SWA **(** **Supplementary Figure 4C** **)**. Overall, these results were further corroborated with the differentially expressed miRNAs in blood T cells of *S. japonicum* infected mice. GO analysis of putative targets of the differentially expressed miRNAs in T cells isolated from blood showed significant enrichment of molecular functions such as protein binding, metal ion binding, nucleotide binding, ATP binding, and DNA binding **(** **Figures 2F–H**; **Supplementary Datasheet 5** **)**. The GO biological processes showed their associations with positive/negative regulation of transcription by RNA polymerase II, multicellular organism development, cell differentiation, *etc.* (**Supplementary Figures 3E–G**; **Supplementary Datasheet 5**).

### MiRNA profiles of T cells from the livers of *S. japonicum* infected mice

**Figure 3:**
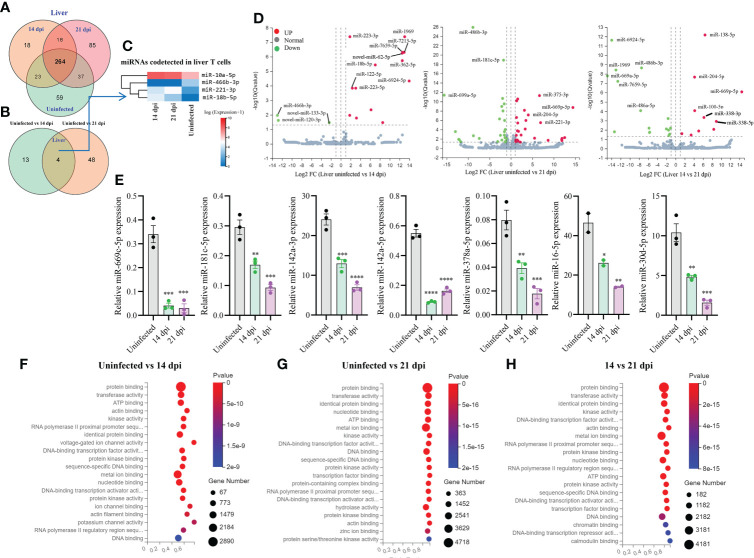
Liver T cell miRNAs profiles, validation of expressions, and prediction of molecular functions of their targets. **(A)** Venn diagram showing total and co-detected liver T cell miRNAs among uninfected, 14 dpi and 21 dpi groups (the number indicates the common or specific miRNAs identified in different groups); **(B)** Venn diagram showing co-detected and specific liver T cell miRNAs between two groups (uninfected vs 14 dpi and uninfected vs 21 dpi); **(C)** Heatmap showing the expression of differentially expressed liver T cell miRNAs co-detected between two groups (uninfected vs 14 dpi and uninfected vs 21 dpi); **(D)** Volcano plot showing differentially expressed liver T cell miRNAs for different comparative analyses (uninfected control vs 14 dpi; uninfected control vs 21 dpi; 14 dpi vs 21 dpi). Green dot indicates down regulated miRNAs, red dot indicates upregulated miRNAs and grey dot indicates no significantly expressed miRNAs; **(E)** Validation of selected increased and decreased expressions of liver T cell miRNAs by RT-qPCR. Data illustrate representative results and show the mean and standard error mean from an experiment carried out in triplicate. Statistical analysis was performed comparing uninfected vs 14 dpi or 21 dpi using one-way ANOVA and * denotes *P* ≤ 0.05, ** denotes *P* ≤ 0.01, *** denotes *P* ≤ 0.001, **** denotes *P* ≤ 0.0001; **(F–H)** GO enrichment analyses of molecular functions of targets for differently expressed miRNAs (Uninfected vs 14 dpi **(F)**; Uninfected vs 21 dpi **(G)**; 14 dpi vs 21 dpi **(H)** in liver T cells). The results showing 20 most significantly enrichment functions of targets for differentially expressed miRNAs.

Totally, 504 miRNAs were detected in T cells isolated from the livers in *S. japonicum* infected and uninfected mice, respectively (**Figure 3A**). Among them, 59 and 85 miRNAs were specifically detected at 21 dpi and uninfected control, respectively, whereas 18 miRNAs were specifically detected at 14 dpi (**Figure 3A**). We observed 264 co-detected miRNAs, of which 52 and 133 miRNAs were up- and downregulated compared to the uninfected control, respectively (**Supplementary Figures 2E, F**). Analysis of the differentially expressed miRNAs between two groups (uninfected vs 14 dpi: 3 decreased and 14 increased; uninfected vs 21 dpi: 26 decreased and 26 increased) showed the co-detected and specific miRNAs (**Figure 3B**; **Supplementary Datasheet 3**). In addition, seven novel miRNAs (*novel-miR-133-3p*, *novel-miR-120-3p*, *novel-miR-62-5p*, *novel-miR-279-3p*, *novel-miR-389-3p*, *novel-miR-226-3p*, and *novel-miR-44-5p*, mark in blue) were also shown to differentially express in T cell of liver between *S. japonicum* infected mice (14 dpi or 21 dpi and uninfected control (**Supplementary Figures 3C, D** **)**. The heatmap of miRNAs in liver T cells at 14 dpi and 21 dpi showed four co-detected differentially expressed miRNAs (*miR-10a-5p*, *miR-466b-3p*, *miR-221-3p* and *miR-18b-5p*) (**Figure 3C**). Volcano plot analysis of differentially expressed miRNAs between uninfected and 14 dpi mice showed the downregulation of *miR-466b-3p*, *novel-miR-120-3p* and *novel-miR-133-3p*, and the upregulation of *miR-223-3/5p*, *miR-1969*, *miR-7213-5p*, *miR-7659-5p*, *novel-miR-62-5p*, *miR-18b-5p*, *miR-362-5p*, *miR-122-5p* and *miR-6924-5p* as well as other 4 miRNAs (**Figure 3D**). In comparison with uninfected, miRNAs such as *miR-486b-3p*, *miR-181c-5p*, *miR-669a-5p* were downregulated in T cells isolated from 21 dpi murine livers, while *miR-375-3p*, *miR-204-5p*, *miR-669p-5p*, *miR-221-3p* and others exhibited notable upregulation (**Figure 3D**). In addition, a panel of miRNAs was found to be differentially expressed in liver T cells between 14 dpi and 21 dpi (**Figure 3D**). The RT-qPCR validations of selected miRNAs indicated *miR-669c-5p*, *miR-181c-5p*, *miR-142a-3p/5p*, *miR-378a-5p*, *miR-16-5p*, and *miR-30d-5p* exhibited significantly decreased expressions in murine liver T cells at 14 dpi and 21 dpi compared to uninfected control (**Figure 3E**). The RT-qPCR results of these altered expressions of T cell miRNAs were consistent (80%) with RNA-seq results. Furthermore, isolated T cells from liver treated with SWA showed increased expressions of *miR-182-5p, miR-21a-5p* and *miR-222-3p* while decreased expressions of *miR-142a-3p, miR-181c-5p, miR-142a-5p, miR-191-5p* and *miR-467a-5p* were observed (**Supplementary Figure 4B**). Similar results were also noted in EL-4 T cell treated with SWA (**Supplementary Figure 4C**). Overall, these results were further corroborated with the differentially expressed miRNAs in liver T cells of *S. japonicum* infected mice. GO enrichment analysis of molecular functions of targets for differentially expressed miRNAs in three groups (uninfected vs 14 dpi; uninfected vs 21 dpi; 14 dpi vs 21 dpi) showed several significant binding functions, including protein binding, metal ion binding, nucleotide binding, ATP binding, DNA binding, *etc.* (**Figures 3F–H**; **Supplementary Datasheet 6**). GO analysis of biological processes of targets for differentially expressed miRNAs in these groups showed their significant enrichment with positive/negative regulation of transcription by RNA polymerase II, multicellular organism development, cell differentiation, *etc.* (**Supplementary Figures 3H-J**; **Supplementary Datasheet 6**). These results suggest that most targets of these differentially expressed miRNAs were associated with binding potentially involved with posttranscriptional gene regulation.

**Figure 4:**
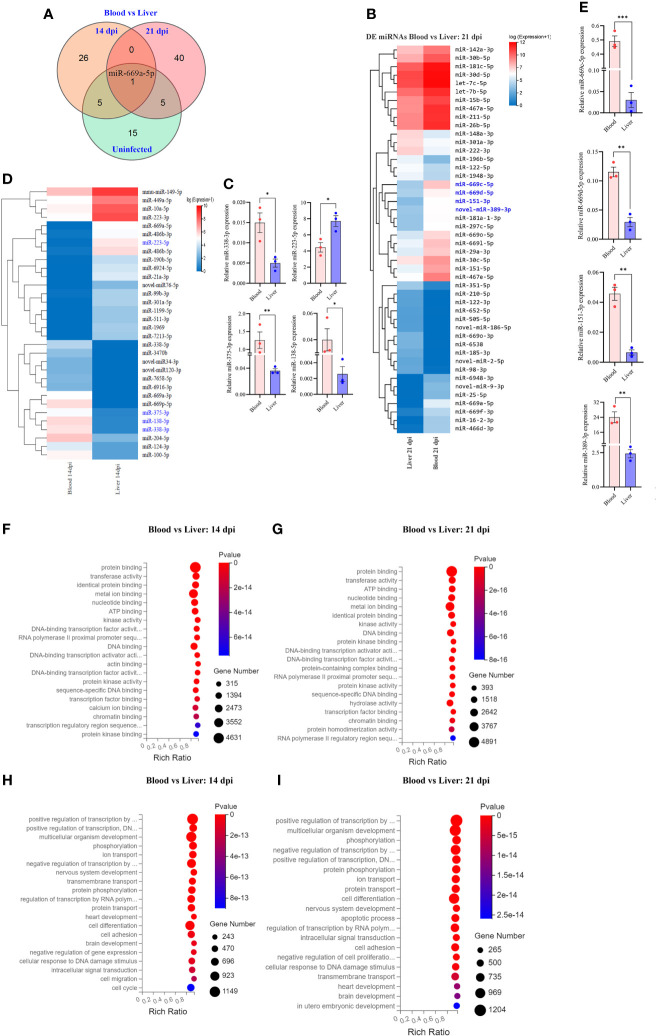
Comparative analyses of T cell miRNA expressions between blood and liver at 14 dpi and 21 dpi. **(A)** Comparison of differentially expressed miRNAs between blood and liver showing co-detected and specific miRNAs at 14 dpi, 21 dpi and uninfected control; **(B)** Heatmap showing differentially expressed T cell miRNAs between blood and liver at 14 dpi (miRNAs highlighted in blue color are validated by RT-qPCR); **(C)** RT-qPCR validation of the expressions of selected miRNAs between blood and liver at 14 dpi. Data illustrate representative results and show the mean and standard error mean from an experiment carried out in triplicate. Statistical analysis was performed comparing blood and liver using Student’s T-test and * denotes *P* ≤ 0.05, ** denotes *P* ≤ 0.01. **(D)** Heatmap showing differentially expressed T cell miRNAs between blood vs liver at 21 dpi (miRNAs highlighted in blue color are validated by RT-qPCR); **(E)** Validation of the expressions of selected miRNAs from blood vs liver at 21 dpi by RT-qPCR; For RT-qPCR, data illustrate representative results and show the mean and standard error mean from an experiment carried out in triplicate. Statistical analysis was performed between *S. japonicum* infected mice blood and liver isolated T cells at 14 dpi and 21 dpi using Students T test and ** denotes *P* ≤ 0.01, *** denotes *P* ≤ 0.001; **(F, G)** GO analysis of molecular functions of targets for differentially expressed T cell miRNAs between blood and liver at 14 dpi **(F)** or at 21 dpi **(G)**; **(H, I)** GO analysis of biological processes of targets for differentially expressed T cell miRNAs between blood and liver at 14 dpi **(H)** or at 21 dpi **(I)**.

### Comparative analysis of differentially expressed miRNAs between blood and liver T cells

Comparative analysis of T cell miRNAs between blood and liver for 14 dpi, 21 dpi and uninfected control indicated 265, 402 and 351 co-detected miRNAs **(** **Supplementary Figures 3K–M**). Whereas differentially expressed miRNAs between liver and blood for 14 dpi, 21 dpi and uninfected control, we found 92 miRNAs and observed one co-detected miRNA (*miR-669a-5p*) (**Figure 4A**; **Supplementary Datasheet 4**). We further compared the expression pattern of these differentially expressed miRNAs of T cells between blood and liver at 14 dpi and visualized by heatmaps (**Figure 4B**). We selected a few miRNAs to validate their expressions between liver and blood of *S. japonicum* infected mice at 14 dpi. The results indicated a higher expression of *miR-223-5p* in T cells isolated from the liver compared to the blood T cell at 14 dpi (**Figure 4C**). In contrast, a significantly decreased expression was observed in *miR-375-3p*, *miR-138-5p* and *miR-338-3p* in liver T cells at 14 dpi (**Figure 4C**). In addition, the expression pattern of differentially expressed miRNAs between blood and liver T cells at 21 dpi was shown in **Figure 4D**. Validation of several selected miRNAs by RT-qPCR showed that the expressions of *miR-669c-5p*, *miR-669d-5p*, *miR-151-3p*, and *novel-miR-389-3p* were significantly increased in blood isolated T cells compared to the liver at 21 dpi (**Figure 4E**). GO molecular function analysis of enriched terms for targets of differentially expressed miRNAs between blood and livers at 14 dpi and 21 dpi showed these targets were potentially associated with protein binding, metal ion binding, nucleotide binding, ATP binding, DNA binding, *etc.* (**Figures 4F, G**; **Supplementary Datasheet 7**). GO analysis of biological processes showed their targets associated with positive and negative regulation of transcription by RNA polymerase II multicellular organism development, cell differentiation and others in case of blood vs liver T cells at 14 dpi and 21 dpi (**Figures 4H, I**; **Supplementary Datasheet 7** **)**. These results suggest that there may be specific functional cues that lead to distinct expression patterns observed between blood and liver isolated T cells.

### RT-qPCR analysis of selected miRNA targets at different stages of *S. japonicum* infection and bioinformatic analysis of regulatory networks

**Figure 5:**
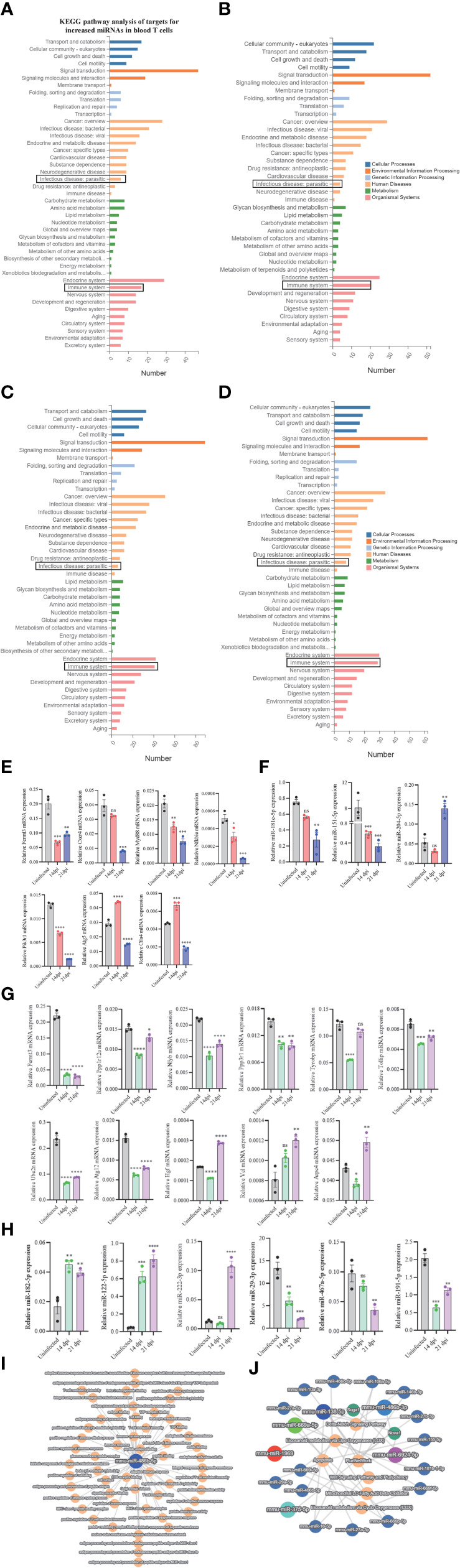
KEGG analyses of targets for differentially expressed miRNAs and RT-qPCR analysis of the expressions of target genes. **(A–D)** KEGG analysis of up- and downregulated miRNA targets (uninfected vs 14 dpi; uninfected vs 21 dpi) in murine blood **(A, B)** or liver **(C, D)** associated with human disease and organismal systems in blood and liver. Rectangle indicates selected miRNAs targets associated the pathways of infectious (parasitic) disease and immune system; **(E)** RT-qPCR analysis of the expressions of target genes for differently expressed miRNAs in blood T cells. The putative targets associated with parasitic disease and immune system were selected for RT-qPCR analysis. Data illustrate representative results and show the mean and standard error mean from an experiment carried out in triplicate. Statistical analysis was performed comparing uninfected vs 14 dpi or 21 dpi using one-way ANOVA and * denotes *P* ≤ 0.05, ** denotes *P* ≤ 0.01, *** denotes *P* ≤ 0.001, **** denotes *P* ≤ 0.0001 and ns denotes non-significant; **(F)** RT-qPCR validation of the expressions of several miRNAs in blood T cells that potentially regulate the corresponding targets as shown in **(E)**. *Pik3r1* and *Nfkbie* are the targets of *miR-204-5p*; *Ctla4* is the target of *miR-151-5p*; *Atg5* is the target of *miR-181c-5p*. Data illustrate representative results and show the mean and standard error mean from an experiment carried out in triplicate. Statistical analysis was performed comparing uninfected vs 14 dpi or 21 dpi using one-way ANOVA and ** denotes *P* ≤ 0.01, *** denotes *P* ≤ 0.001 and ns denotes non-significant. **(G)** RT-qPCR analysis of the expressions of target genes for differently expressed miRNAs in liver T cells. The putative targets associated with parasitic disease and immune system were selected for RT-qPCR analysis. Data illustrate representative results and show the mean and standard error mean from an experiment carried out in triplicate. Statistical analysis was performed comparing uninfected vs 14 dpi or 21 dpi using one-way ANOVA and * denotes *P* ≤ 0.05, ** denotes *P* ≤ 0.01, *** denotes *P* ≤ 0.001, **** denotes *P* ≤ 0.0001 and ns denotes non-significant; **(H)** RT-qPCR validation of the expressions of several miRNAs in liver T cells that potentially regulate the corresponding targets as shown in **(G)**. *Ppp3r1 and Tollip* are the targets of *miR-182-5p*; *Nfyb* and *Ppp1r12a* are the target of *miR-222-3p*; *Arpc4* is the target of *miR-191-5p*; *Fermt3* is the target of *miR-122-5p*; *Vcl* is the target of *miR-467a-5p*; *Hgf* is a target of *miR-29a-3p*. Data illustrate representative results and show the mean and standard error mean from an experiment carried out in triplicate. Statistical analysis was performed comparing uninfected vs 14 dpi or 21 dpi using one-way ANOVA and ** denotes *P* ≤ 0.01, *** denotes *P* ≤ 0.001 and ns denotes non-significant. **(I)** *miR-486b-5p* decreased in blood T cells as shown in **Figure 2E** potentially regulates many biological processes by interacting with its corresponding targets; **(J)** Top differentially expressed T cell miRNAs including *miR-486b-5p/3p*, *miR-375-5p*, *miR-1969*, and *miR-6924-5p* are putatively involved in the regulations of Wnt signaling pathway and pluripotency, Delta-Notch signaling pathway, mitochondrial LC-fatty acid beta-oxidation, eicosanoid metabolism *via* cyclo oxygenases (COX) and others by interacting with their targets.

To assess the regulatory roles of T cell miRNAs, we evaluated the expressions of several selected miRNA targets that are predicted to be associated with the KEGG pathways associated immune system and parasite-caused infectious diseases **(** **Figures 5A–D**; **Supplementary Datasheet 8** **)**. We noted several miRNAs targets potentially associated with parasitic infections and T cell immune response during these stages of infection. RT-qPCR analyses of several selected miRNA targets potentially involved in parasitic infections and T cell immune response **(** **Figure 5E** **)** and their corresponding miRNA expressions **(** **Figure 5F** **)** at blood T cells showed a generally negative correlation, suggesting that the miRNAs could regulate their targets at blood T cells during *S. japonicum* infection. For example, the expressions of targets such as cytotoxic T-lymphocyte-associated protein 4 (*Ctla4*, a target of downregulated *miR-151-5p*) and autophagy-related 5 (*Atg5*) (a target of downregulated *miR-181c-5p*) were increased in blood T cells at 14 dpi compared to uninfected control while the expressions of *miR-151-5p* and *miR-181c-5p* were decreased (**Figures 5E** **, F**). Similar results were also observed at liver T cells between the expressions of target genes (**Figure 5G**) and their corresponding miRNAs (**Figure 5H**). Similarly, the liver isolated T cells target genes such as hepatocyte growth factor (*Hgf*, a target of downregulated *miR-29-3p*), vinculin (*Vcl*, a target of downregulated *miR-467a-5p*) and actin-related protein 2/3 complex, subunit 4 (*Arpc4*, a target of downregulated *miR-191-5p*) shown increased expression at liver T cell of 21 dpi, whereas the *Fermt3* (a target of upregulated *miR-122-5p*), protein phosphatase 1, regulatory subunit 12A (*Ppp1r12a)* and nuclear transcription factor-Y beta (*Nfyb*) (targets of upregulated *miR-222-3p*), toll interacting protein (*Tollip*) and protein phosphatase 3 regulatory subunit B, Beta (*Ppp3r1*) (targets of upregulated *miR-182-5p*) shown decreased expression in liver T cells following *S. japonicum* infection (**Figures 5G** **, H**). Additionally, several selected differentially expressed miRNAs at 14 dpi or 21 dpi compared to uninfected control (decreased in blood and liver: *miR-486b-5p/3p*, *miR-6924-5p*; increased in blood and liver: *miR-375-5p* and *miR-1969*) suggested that these miRNAs are related with diverse functions associated with infection. In particular, *miR-486b-5p* shown decreased expression in *S. japonicum* infected mice at 14 dpi and 21 dpi compared to uninfected control (**Figure 2E**) and is putatively associated with adaptive immune response, peptide binding, T cell-mediated immune response, nuclear outer membrane-endoplasmic reticulum membrane network, antigen binding, protein-containing complex binding, peptide antigen binding, endoplasmic reticulum membrane, and others (**Figure 5I**). Similarly, differentially expressed miRNAs (downregulated: *miR-486b-3p*, *miR-6924-5p*; upregulated: *miR-1969*, *miR-375-5p*, *miR-669a-5p*, and *miR-138-5p*) were shown to be putatively involved in the regulations of apoptosis, Wnt signaling pathway and pluripotency, eicosanoid metabolism *via* cyclooxygenase (COX), eicosanoid metabolism *via* lipo oxygenase (LOX) and mitochondrial LC-fatty acid beta-oxidation (**Figure 5J**).

## Discussion

T cells are one of most important lymphocytes and the main elements of adaptive immunity. The potential role of miRNAs in regulating T cell proliferation, activation, and differentiation has been well discussed (24, 45). However, the expression profiles of host T cell miRNAs during *Schistosoma* infection are scanty. The liver stages are critical for schistosomula development and schistosome-caused pathology. *Schistosoma* are blood dwelling flatworms. Blood acts as a pipeline for immune system, carry different immune cells from one place to another and respond according to the types of infections. Most of the studies on schistosomes have carried out on the adult worm infection stages to observed mostly eggs induced immune response. However, very few studies have focused on early stages especially on schistosomula and pre-egg laying worms, which are important for worm development and maturation for finally residing. In addition, schistosomula are also considered to be a valuable stage for vaccine development to dump worm parasitism. Therefore, we undertook the study to profile the miRNAs repertoire in peripheral blood and livers to understand T cell miRNA alteration that may be involved in T cell-mediated immune response during the parasitic infection.

*Schistosoma* infection induces different immune responses. In the early phase of infection, cercaria initiates the Th1 immune response, and the produced eggs induce a shift towards Th2 type immune response (11, 12). The Th2 immune response plays an important role in the pathogenesis of schistosomiasis (46). At 14 and 21 dpi, we observed a significant decrease in the expression of *miR-486b-5p/3p*, *miR-122-5p*, *miR-181c-5p* and *miR-6924-5p etc.* In contrast, a significant increase in the expression of miRNAs such as *miR-375-5p*, *miR-466c-3p*, and *miR-138-5p* in both blood and livers isolated T cells. The functional aspect of some of these miRNAs has been documented previously; however, their specific roles during *S. japonicum* infection remain unknown. Bioinformatic analysis of top differentially expressed miRNAs and their targets suggested their putative roles in infections and immune responses, especially adaptive immune responses (**Figures 5I** **, G**). GO analysis of targets of differentially expressed miRNAs suggested that most of the miRNAs may be involved in signal transduction, signal molecule and interactions, infectious disease and others.

We observed the increased expression of *Ctla4* and *Atg5* of growth signal transduction protein kinases in blood (**Figure 5E**). T cells circulate consistently between blood, lymphoid tissue, and lymph nodes to encounter foreign antigens presented by DCs (47). Ctla4 limits the interactions of CD4^+^ T-cells with DCs by modulating the threshold for T cell activation and induces T cell motility response in secondary lymphoid organs (48). The other target gene, Atg5, has been shown to be responsible for the activation and differentiation of innate and adaptive immune cells and then promotes the interaction between T cells or B cells and antigen-presenting cells (49). The increased expression of these target genes (*Ctla4* and *Atg5*) during schistosome infection at 14 dpi, especially during blood stages, may potentially involve in T cells mediated immune response. Hgf is a pleiotropic cytokine that influences mitogenesis, motility and differentiation of many different cell types (50). It also maintains the differentiation of hepatic sinusoidal endothelial cells, specializing in lymphocyte recruitment to the liver (51). The increased expression of *Hgf* in liver at 21 dpi may suggested the organ specific immune response during *S. japonicum* infection. Actin-related protein is highly conserved in eukaryotes that nucleate branched actin filaments and generate actin networks (52). It includes five subunits and Arpc2, and 4 forms the core of this complex, and the deficiency in Arpc led to the decrease in the number of peripheral T cell (53). The increased expression of *Arpc4* may suggest to potentially regulate T cells populations during *S. japonicum* infection.

Considering the important roles of miRNAs in T cell development program and function, several groups have documented miRNA profiles in different types of T cells (54–56). We observed a differentially decreased expression of *miR-486b-5p/3p* in T cells isolated from blood and liver. *miR-486a-5p* and *miR-486b-5p* originate from the different pre-miRNAs transcribed from the opposite strand of the same genomic locus; however, they share the same mature sequences (57). Primarily *miR-486-5p* was identified as a tumor-suppressive miRNA in lung cancer (58), and shown decreased expression in breast cancer patients (59). Another study suggested that the inhibition of *miR-486-5p* alleviated LPS-induced cell damage by limiting inflammatory injury, oxidative stress and apoptosis by targeting NRF1 (60). The decreased expression of *miR-486* in the present study may lead to minimal inflammatory response at 14 dpi and 21 dpi of *S. japonicum* infection. *miR-223* is one of the differentially expressed miRNAs identified in our study that has been shown to play a vital role in the immune response, regulating multiple processes from myeloid differentiation to neutrophil, macrophage, and DC function (61). The changes of *miR-223-3p* expression are linked to macrophage apoptosis (62) and play an essential role in maintaining the balance of innate immunity to avoid excess inflammation and tissue damage. Another study indicated that *miR-223* level is negatively associated with lymphocyte apoptosis by targeting FOXO1 during sepsis (63). Further investigation of the roles of these differently expressed miRNAs may gain important insight into how miRNAs involve in immune response during *S. japonicum* infection, then resulting into the development of effective strategies for schistosomiasis control.

Comparative analysis of the identified miRNAs between blood and liver at 14 dpi or 21 dpi, we noted the majority of differently expressed miRNAs was specially associated with *S. japonicum* infection at 14 dpi or 21 dpi (**Figure 4A**). However, there were a few of differently expressed miRNAs between blood and livers showing to be co-detected between 14 dpi/21 dpi and uninfected controls (**Figure 4A**). For example, miRNAs such as *miR-10a-5p, miR-149-5p, miR-223-3p, miR-669a-3p* and *miR-669p-5p* showed to be co-detected for different expressions between blood and livers among 14 dpi and uninfected control and *miR-122-5p*, *miR-142a-3p*, *miR-151-5p, miR-211-5p* and *miR-15b-5p* showed to differently express in both 21 dpi and uninfected control. Interestingly, we observed an increased expression of *miR-669a-5p* in T cells isolated from murine blood during *Schistosoma* infection (14 dpi vs 21 dpi) while a decreased expression of this miRNA in T cells isolated from liver was observed. The results suggested that *miR-669a-5p* may have different roles in different organs during *Schistosoma* infection. *miR-122* levels have been suggested to be a diagnostic marker for liver disease. The decreased expression of *miR-122* in T cells isolated from blood and liver of *S. japonicum*-infected mice may potentially be associated with increased adaptive immune response and decreased innate immunity since the increased level of *miR-122* was shown to link with hepatocyte innate immunity (64). Comparative analysis of T cell miRNA profiles between blood and liver shows dynamic expression patterns. Among them, we noted *miR-669a-5p* was shown to be the common differentially expressed between blood and livers. Unfortunately, the role of *miR-669a* in immune response remain unknown although a study suggested its role in the prevention of skeletal muscle differentiation and in postnatal cardiac progenitors (65). Consequently, it is worth to investigate whether *miR-669a-5p* regulates T cell response during the early stage of *S. japonicum* infection.

In conclusion, our study presents a comprehensive dataset of differentially expressed T cell miRNAs from blood and liver of *S. japonicum* infected mice at differently early hepatic schistosomula stages. Several panels of differentially expressed miRNAs, such as *miR-486a-5p/3p*, *miR-486b-5p/3p*, *miR-375-3p*, *miR-466a-5p/3p*, *miR-466b-5p/3p*, *miR-223-3p*, *miR-181c-5p*, *etc.*, were identified to be putatively associated with T cell immune response showing dynamic expressions during *S. japonicum* infection. Further studies unpinning the potential role of these miRNAs are expected to provide the translational value for understanding and application of miRNAs mediated T cell immune response during *Schistosoma* infection.

## Data availability statement

The data presented in the study are deposited in China National GeneBank DataBase, accession number CNP0003350.

## Ethics statement

The animal study was reviewed and approved by The Animal Management Committee and the Animal Care and Use Committee of the Shanghai Science and Technology Commission of the Shanghai Municipal government for Shanghai Veterinary Research Institute, Chinese Academy of Agriculture Sciences, China.

## Author contributions

Conceptualization, BG and GC; investigation, SL, BG, and LQ; writing original draft, BG; Supervision, GC; review and editing, BG, GC, CF, SY, and MP. All authors contributed to the article and approved the submitted version.

## Funding

This study was supported, in whole or in part, by the Key Program for International S&T Cooperation Projects of China (2021YFE0191600 to GC), the State Key Laboratory of Veterinary Etiological Biology (SKLVEB2020KFKT018 to GC), the Research Fund for International Young Scientists from NNSF (31950410564 to BG) and the National Natural Science Foundation of China (31472187 and 31672550 to GC). The funders had no role in study design, data collection, analysis, decision to publish, or manuscript preparation.

## Conflict of interest

The authors declare that the research was conducted in the absence of any commercial or financial relationships that could be construed as a potential conflict of interest.

## Publisher’s note

All claims expressed in this article are solely those of the authors and do not necessarily represent those of their affiliated organizations, or those of the publisher, the editors and the reviewers. Any product that may be evaluated in this article, or claim that may be made by its manufacturer, is not guaranteed or endorsed by the publisher.

## Supplementary material

Supplementary Figure 1 Flow cytometry sorting of T cells and cell viability analysis. **(A, B)** Representative flow cytometry sorting of T cells isolated from blood **(A)** and liver **(B)** of *S. japonicum* infected mice and uninfected control; **(C, D)** Flow cytometry analysis of percentage of live T cells isolated from the blood **(C)** and liver **(D)** of *S. japonicum* infected mice and uninfected control; **(E, F)** Flow cytometry analysis of percentage of CD4^+^ and CD8a^+^ cells isolated from the blood **(E)** and liver **(F)** of *S. japonicum* infected mice and uninfected control. Representative results presented as the average from two biological replicates.

Supplementary Figure 2 Classifications of distributions of small RNAs identified by RNA seq and heatmap showing differently expressed miRNAs. **(A)** Classifications of distributions of small RNAs identified from T cells isolated from murine blood for each library; **(B)** Classifications of distributions of small RNAs identified from T cells isolated from murine livers for each library; **(C)** Heatmap showing increased expressions of miRNAs in blood T cells; **(D)** Heatmap showing decreased expressions of miRNAs in blood T cells; **(E)** Heatmap showing increased expressions of miRNAs in liver T cells; **(F)** Heatmap showing decreased expressions of miRNAs in liver T cells; **(G)**. Heatmap of miRNAs expression showing increased miRNAs such as *miR-10a-5p* in uninfected liver vs uninfected blood T cells. Red color showed higher expression and blue color showed lower expression.

Supplementary Figure 3 Differentially expressed miRNAs from blood/liver T cells between *S. japonicum* infected mice and uninfected control and predictions of the biological processes of their targets. **(A)** Heatmap showing differentially expressed miRNAs from blood T cells between uninfected control and *S. japonicum* infected mice at 14 dpi**; (B)** Heatmap showing differentially expressed miRNAs from blood T cells between uninfected control and *S. japonicum* infected mice at 21 dpi; **(C)** Heatmap showing differentially expressed miRNAs from liver T cells between uninfected control and *S. japonicum* infected mice at 14 dpi; **(D)** Heatmap showing differentially expressed miRNAs from liver T cells between uninfected control and *S. japonicum* infected mice at 21 dpi. **(E)** GO analyses of biological processes of the targets for differentially expressed miRNAs (uninfected vs 14 dpi) from blood T cells; **(F)** GO analyses of biological processes of the targets for differentially expressed miRNAs (uninfected vs 21 dpi) from blood T cells; **(G)** GO analyses of biological processes of the targets for differentially expressed miRNAs (14 dpi vs 21 dpi) from blood T cells; **(H)** GO analyses of biological processes of the targets for differentially expressed miRNAs (uninfected vs 14 dpi) from liver T cells; **(I)** GO analyses of biological processes of the targets for differentially expressed miRNAs (uninfected vs 21 dpi) from liver T cells; **(J)** GO analyses of biological processes of the targets for differentially expressed miRNAs (14 dpi vs 21 dpi) from blood T cells; **(K-M)** Comparative analysis of differently expressed T cell miRNAs for each biological replicate between blood and liver at 14 dpi **(K)**, 21 dpi **(L)** and uninfected **(M)**.

Supplementary Figure 4 RT-qPCR analysis of selected miRNA expressions in isolated T cells and *in vitro* cultured EL-4 cells treated with *S. japonicum* worm antigens (SWA). **(A)** RT-qPCR analysis of the expressions of selected miRNAs in blood T cells isolated from mice treated with or without SWA; **(B)** RT-qPCR analysis of the expressions of selected miRNAs in liver T cells isolated from T cells treated with or without SWA; **(C)** RT-qPCR analysis of the expressions of selected miRNAs in EL-4 cells treated with or without SWA. Data illustrate representative results and show the mean and standard error mean from an experiment carried out in triplicate. Statistical analysis was performed on T cells from blood and liver and EL-4 cells between SWA treatment and controls using Student’s T-test and * denotes *P* ≤ 0.05, ** denotes *P* ≤ 0.01, *** denotes *P* ≤ 0.001. **** denotes *P* ≤ 0.0001.

Supplementary Data Sheet 1 T cells raw reads filtration.

Supplementary Data Sheet 2 T cells clean reads mapping.

Supplementary Data Sheet 3 The list of differentially expressed T cell miRNAs in blood or liver among uninfected control, 14 dpi and 21 dpi.

Supplementary Data Sheet 4 The list of differently expressed T cell miRNAs between blood and livers in uninfected control, 14 dpi and 21 dpi.

Supplementary Data Sheet 5 The list of GO enrichment analysis of target mRNAs of differentially expressed miRNAs in blood.

Supplementary Data Sheet 6 The list of GO enrichment analysis of target mRNAs of differentially expressed miRNAs in liver.

Supplementary Data Sheet 7 The list of GO enrichment analysis of target mRNAs for differentially expressed miRNAs between blood and liver.

Supplementary Data Sheet 8 The list of KEGG pathway analysis target genes for increased/decreased miRNAs in blood/liver T cells.

Supplementary Table 1 Details of antibodies used for isolating T cells from liver and blood of mice.

Supplementary Table 2 List of RT-qPCR primers used to validate T cell miRNA expressions.

Supplementary Table 3 List of primers used for analyzing the expressions of miRNA targets by RT-qPCR.
