# Supplementary figures and images for: Dynamic miRNA profile of host T cells during early hepatic stages of Schistosoma japonicum infection
Source: Front Immunol. 2022 Sep 2;13:911139. doi: 10.3389/fimmu.2022.911139 (PMC9478579; doi:10.3389/fimmu.2022.911139)

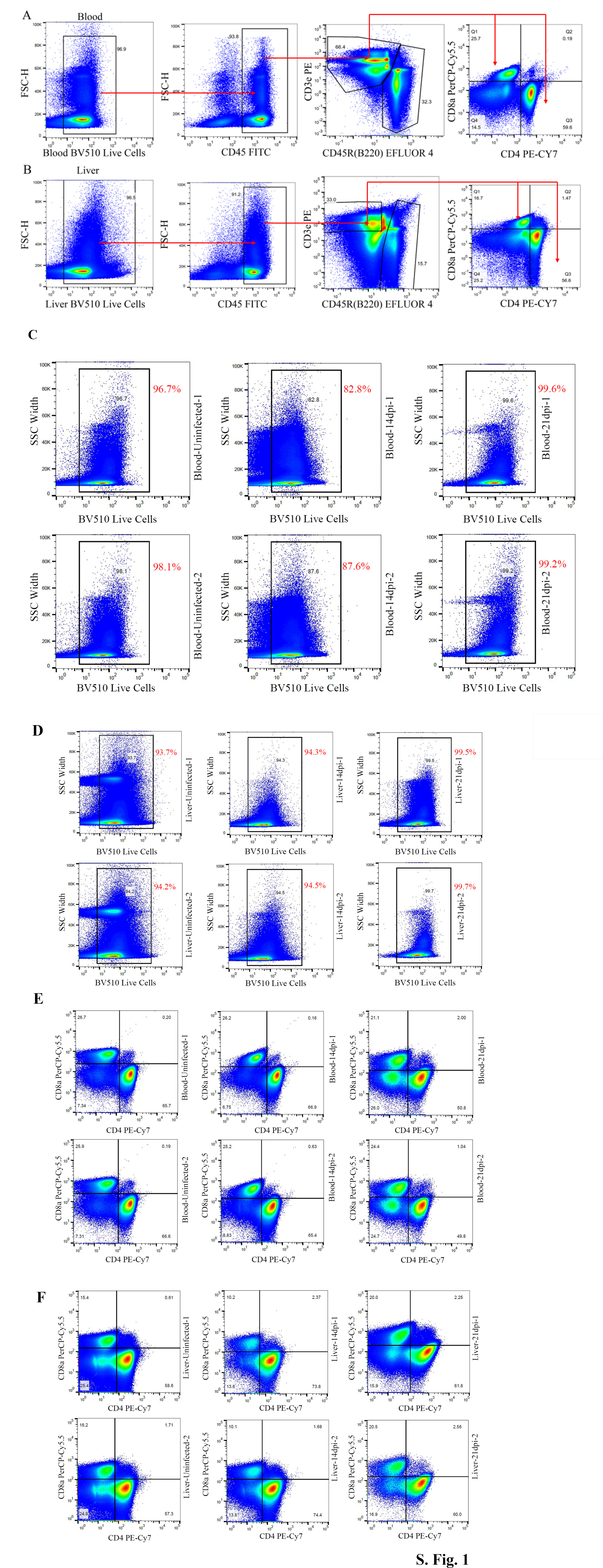

Supplement: Supplementary Figure 1 — Flow cytometry sorting of T cells and cell viability analysis. (A, B) Representative flow cytometry sorting of T cells isolated from blood (A) and liver (B) of S. japonicum infected mice and uninfected control; (C, D) Flow cytometry analysis of percentage of live T cells isolated from the blood (C) and liver (D) of S. japonicum infected mice and uninfected control; (E, F) Flow cytometry analysis of percentage of CD4+ and CD8a+ cells isolated from the blood (E) and liver (F) of S. japonicum infected mice and uninfected control. Representative results presented as the average from two biological replicates. [file Image_1.jpeg]

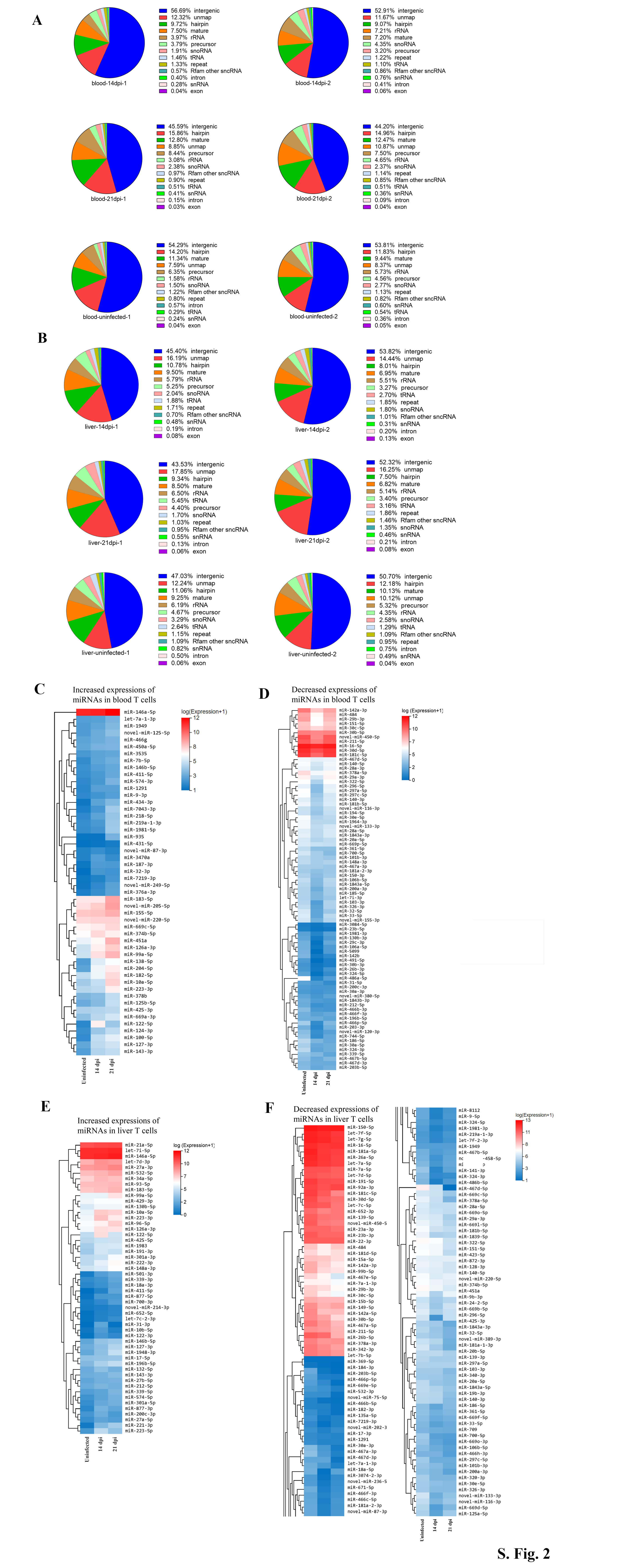

Supplement: Supplementary Figure 2 — Classifications of distributions of small RNAs identified by RNA seq and heatmap showing differently expressed miRNAs. (A) Classifications of distributions of small RNAs identified from T cells isolated from murine blood for each library; (B) Classifications of distributions of small RNAs identified from T cells isolated from murine livers for each library; (C) Heatmap showing increased expressions of miRNAs in blood T cells; (D) Heatmap showing decreased expressions of miRNAs in blood T cells; (E) Heatmap showing increased expressions of miRNAs in liver T cells; (F) Heatmap showing decreased expressions of miRNAs in liver T cells; (G). Heatmap of miRNAs expression showing increased miRNAs such as miR-10a-5p in uninfected liver vs uninfected blood T cells. Red color showed higher expression and blue color showed lower expression. [file Image_2.jpeg]

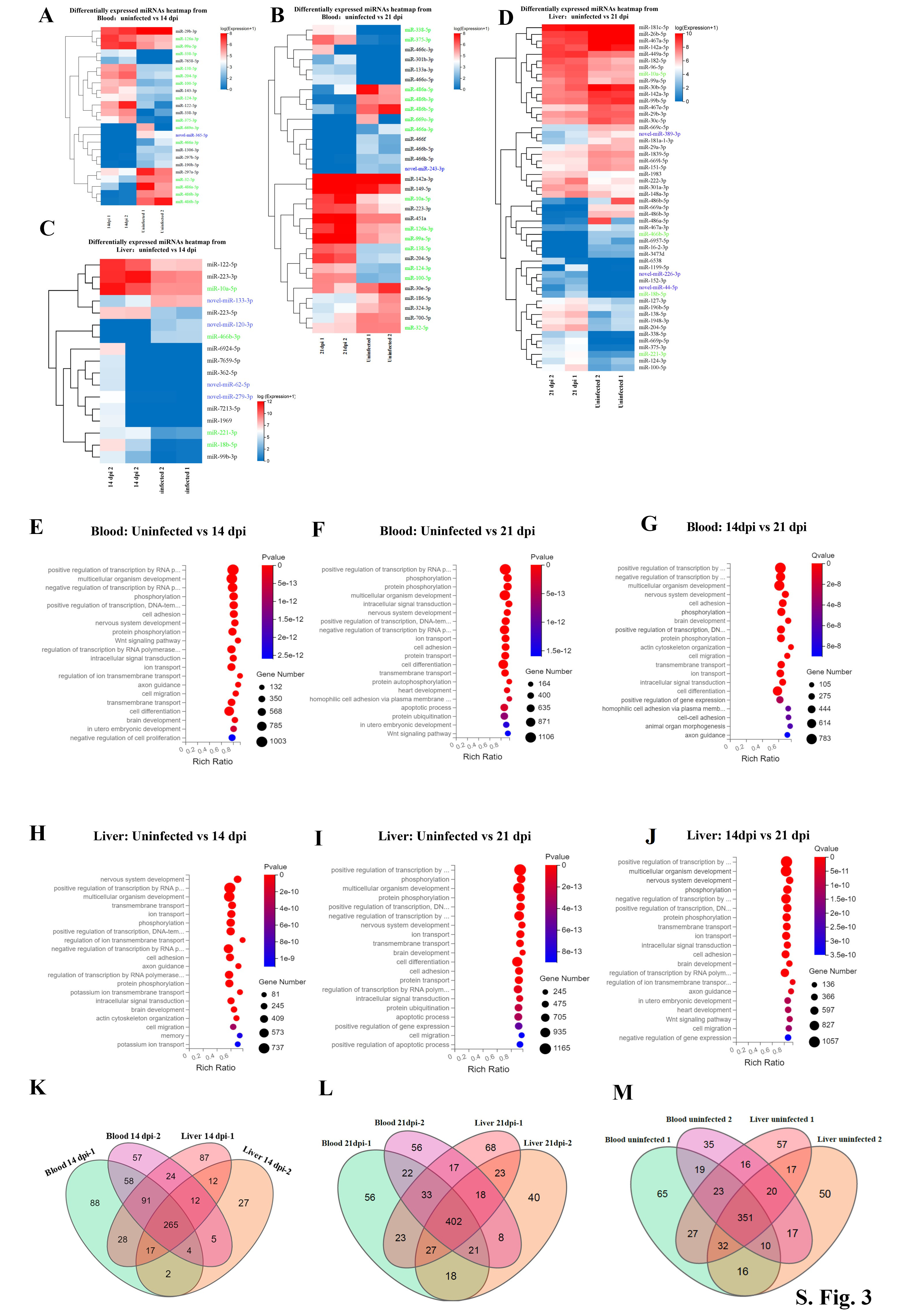

Supplement: Supplementary Figure 3 — Differentially expressed miRNAs from blood/liver T cells between S. japonicum infected mice and uninfected control and predictions of the biological processes of their targets. (A) Heatmap showing differentially expressed miRNAs from blood T cells between uninfected control and S. japonicum infected mice at 14 dpi; (B) Heatmap showing differentially expressed miRNAs from blood T cells between uninfected control and S. japonicum infected mice at 21 dpi; (C) Heatmap showing differentially expressed miRNAs from liver T cells between uninfected control and S. japonicum infected mice at 14 dpi; (D) Heatmap showing differentially expressed miRNAs from liver T cells between uninfected control and S. japonicum infected mice at 21 dpi. (E) GO analyses of biological processes of the targets for differentially expressed miRNAs (uninfected vs 14 dpi) from blood T cells; (F) GO analyses of biological processes of the targets for differentially expressed miRNAs (uninfected vs 21 dpi) from blood T cells; (G) GO analyses of biological processes of the targets for differentially expressed miRNAs (14 dpi vs 21 dpi) from blood T cells; (H) GO analyses of biological processes of the targets for differentially expressed miRNAs (uninfected vs 14 dpi) from liver T cells; (I) GO analyses of biological processes of the targets for differentially expressed miRNAs (uninfected vs 21 dpi) from liver T cells; (J) GO analyses of biological processes of the targets for differentially expressed miRNAs (14 dpi vs 21 dpi) from blood T cells; (K-M) Comparative analysis of differently expressed T cell miRNAs for each biological replicate between blood and liver at 14 dpi (K), 21 dpi (L) and uninfected (M). [file Image_3.tif]

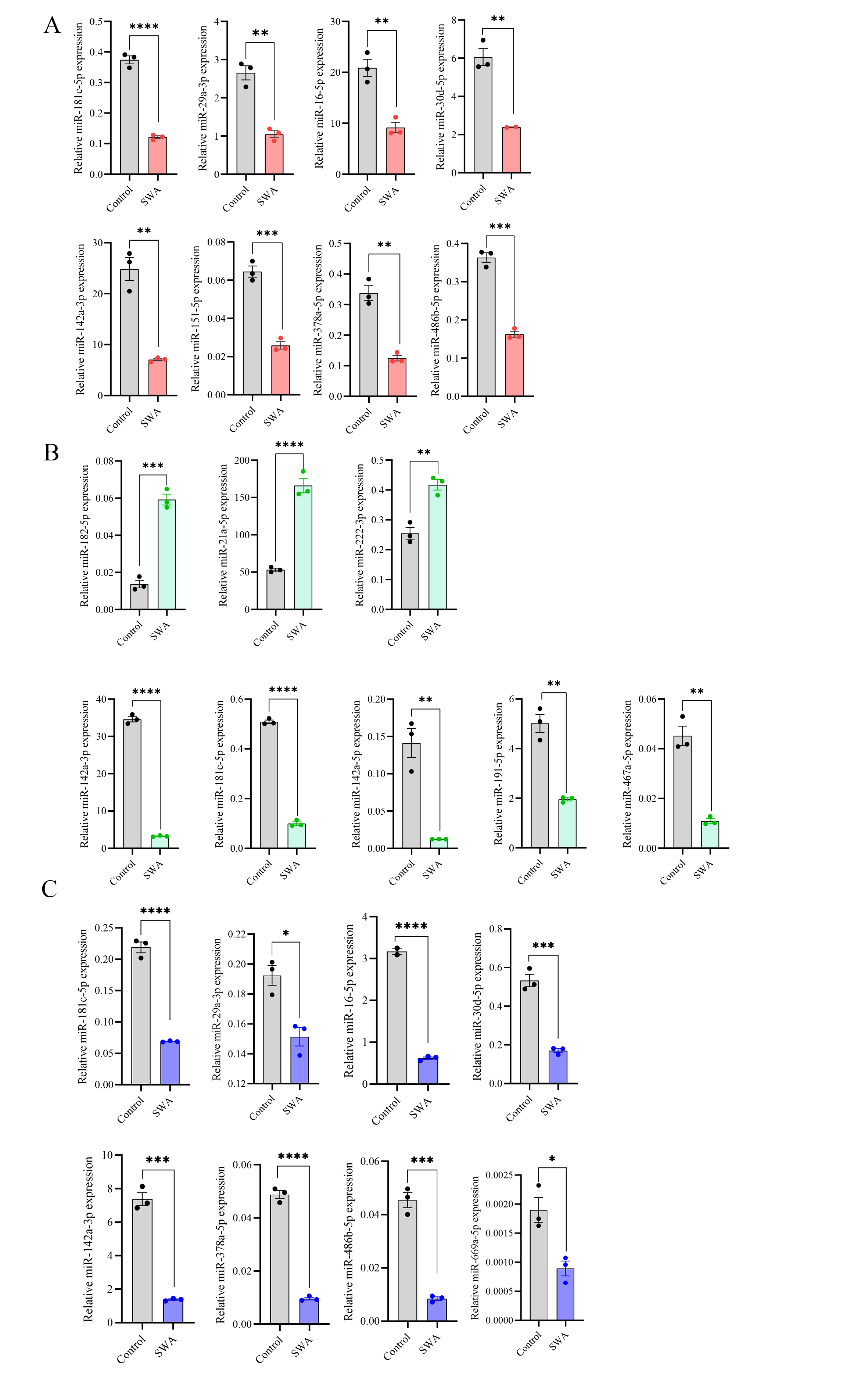

Supplement: Supplementary Figure 4 — RT-qPCR analysis of selected miRNA expressions in isolated T cells and in vitro cultured EL-4 cells treated with S. japonicum worm antigens (SWA). (A) RT-qPCR analysis of the expressions of selected miRNAs in blood T cells isolated from mice treated with or without SWA; (B) RT-qPCR analysis of the expressions of selected miRNAs in liver T cells isolated from T cells treated with or without SWA; (C) RT-qPCR analysis of the expressions of selected miRNAs in EL-4 cells treated with or without SWA. Data illustrate representative results and show the mean and standard error mean from an experiment carried out in triplicate. Statistical analysis was performed on T cells from blood and liver and EL-4 cells between SWA treatment and controls using Student’s T-test and * denotes P ≤ 0.05, ** denotes P ≤ 0.01, *** denotes P ≤ 0.001. **** denotes P ≤ 0.0001. [file Image_4.tif]
