# Supplementary material for: Dynamic miRNA profile of host T cells during early hepatic stages of Schistosoma japonicum infection
Source: Front Immunol. 2022 Sep 2;13:911139. doi: 10.3389/fimmu.2022.911139 (PMC9478579; doi:10.3389/fimmu.2022.911139)
Supplement: Supplementary Table 1 — Details of antibodies used for isolating T cells from liver and blood of mice. [file Table_1.docx]

| **Antibodies** | **Product code** | **CAS No.** | **Company** |
| --- | --- | --- | --- |
| ANTI-MOUSE CD3E (145-2C11) | PE 50 UG | 12-0031-81 | eBioscience |
| [ANTI-MOUSE CD45 (30-F11)](javascript:;) | FITC 50 UG | 11-0451-81 | eBioscience |
| Fixable Viability Stain 510 | / | 564406 | BD |
| ANTI-MOUSE CD45R (RA3-6B2，B220) | EFLUOR 4 | 48-0452-80 | eBioscience |
| ANTI-MOUSE CD4 (GK1.5) | PE-CYANINE | 25-0041-81 | eBioscience |
| ANTI-MOUSE CD8A (53-6.7) | PERCP-CYAN | 45-0081-80 | eBioscience |

**S. Table 1.** Details of antibodies used for isolating T cells from liver and blood of mice.
