# Supplementary material for: Dynamic miRNA profile of host T cells during early hepatic stages of Schistosoma japonicum infection
Source: Front Immunol. 2022 Sep 2;13:911139. doi: 10.3389/fimmu.2022.911139 (PMC9478579; doi:10.3389/fimmu.2022.911139)
Supplement: Supplementary Table 2 — List of RT-qPCR primers used to validate T cell miRNA expressions. [file Table_2.docx]

1. **Table 2. List of RT-qPCR primers used to validate mouse blood and liver isolated T cells.**

| **miRNA** | **Primers (5'-3')** |
| --- | --- |
| miR-99a-3p | AACCCGTAGATCCGATCTTGTG |
| miR-486b-5p | TCCTGTACTGAGCTGCCCCGAG |
| miR-378a-5p | CTCCTGACTCCAGGTCCTGTGT |
| miR-16-5p | TAGCAGCACGTAAATATTGGCG |
| miR-181c-5p | AACATTCAACCTGTCGGTGAGT |
| miR-151-5p | TCGAGGAGCTCACAGTCTAGT |
| miR-30d-5p | TGTAAACATCCCCGACTGGAAG |
| miR-182-5p | TTTGGCAATGGTAGAACTCACACCG |
| miR-204-5p | TTCCCTTTGTCATCCTATGCCT |
| miR-222-3p | AGCTACATCTGGCTACTGGGTCT |
| miR-122-5p | TGGAGTGTGACAATGGTGTTT |
| miR-467a-5p | TAAGTGCCTGCATGTATATGCG |
| miR-191-5p | CAACGGAATCCCAAAAGCAGCTG |
| miR-21a-5p | TAGCTTATCAGACTGATGTTGA |
| miR-669c-5p | ATAGTTGTGTGTGGATGTGTGT |
| miR-142a-3p | TGTAGTGTTTCCTACTTTATGGA |
| miR-142a-5p | CATAAAGTAGAAAGCACTACT |
| miR-29-3p | TAGCACCATCTGAAATCGGTTA |
| miR-223-3p | TGTCAGTTTGTCAAATACCCCA |
| miR-10a-5p | TACCCTGTAGATCCGAATTTGTG |
| miR-486b-3p | CGGGGCAGCTCAGTACAGGA |
| miR-375-3p | TTTGTTCGTTCGGCTCGCGTGA |
| miR-138-5p | AGCTGGTGTTGTGAATCAGGCCG |
| miR-338-3p | TCCAGCATCAGTGATTTTGTTG |
| miR-669a-5p | AGTTGTGTGTGCATGTTCATGTCT |
| miR-669d-5p | ACTTGTGTGTGCATGTATATGT |
| miR-151-3p | CTAGACTGAGGCTCCTTGAGG |
| Novel-miR-389-3p | GCCGAAAGCATGGGAACAGCC |
| U6-F | CTCGCTTCGGCAGCACA |
