# Supplementary material for: Dynamic miRNA profile of host T cells during early hepatic stages of Schistosoma japonicum infection
Source: Front Immunol. 2022 Sep 2;13:911139. doi: 10.3389/fimmu.2022.911139 (PMC9478579; doi:10.3389/fimmu.2022.911139)
Supplement: Supplementary Table 3 — List of primers used for analyzing the expressions of miRNA targets by RT-qPCR. [file Table_3.docx]

**S. Table 3. List of primers used for analysing the expressions of miRNA targets by RT-qPCR**

| **Genes** | **Gene ID** | **Primers (5'-3')** |
| --- | --- | --- |
| *Fermt3* | ENSMUSG00000024965 | Forward: ATGGCGGGTATGAAGACAGC  Reverse: CACCAATGTGCGACTCCCC |
| *Ctla4* | ENSMUSG00000026011 | Forward: CCAGTCTTCTCTGAAGCCATAC  Reverse: GACCTCATCAGTGTTGTGTGA |
| *Atg5* | ENSMUSG00000038160 | Forward: GCGGTTGAGGCTCACTTTAT  Reverse: CAGAGCTGCTTGTGGTCTTT |
| *Pik3r1* | ENSMUSG00000041417 | Forward: CTTGGAGATGATCGACGTACAC  Reverse: GCTCTGTAGTTCTTGGGCTAAA |
| *Myd88* | ENSMUSG00000032508 | Forward: TCCGGCAACTAGAACAGACA  Reverse: AGAAACAACCACCACCATGC |
| *Nfkbie* | ENSMUSG00000023947 | Forward: GAGGCGCTCACATACATCTC  Reverse: GGAAAGCCAAGCAACAGAATAG |
| *Hgf* | ENSMUSG00000028864 | Forward: GGACCATGTGAGGGAGATTATG  Reverse: ATACCAGGACGATTTGGGATG |
| *Vcl* | ENSMUSG00000021823 | Forward: GGCAGAGGTAGTGGAAACTATG  Reverse: CTCCTGCTGTCTCTCATCAATC |
| *Arpc4* | ENSMUSG00000079426 | Forward: GAAGCAGGCCGATGAAATTG  Reverse: GTCATATCCCTCCACAGGTTTC |
| *Ppp1r12a* | ENSMUSG00000019907 | Forward: GTCAGAGGCAGAGACAGATAAG  Reverse: CCTGGTTGGAAGACAGAGTAG |
| *Nfyb* | ENSMUSG00000020248 | Forward: GAGCCTCTGAAACTGTACCTTC  Reverse: CTCTTCGCTTAGTCCATCTGTG |
| *Atg12* | ENSMUSG00000032905 | Forward: TGAAGGCTGTAGGAGACACT  Reverse: AGGCCACCAGTTTAAGGAAC |
| *Ube2n* | ENSMUSG00000074781 | Forward: CAGTTCTGCTATCAATCCAGGC  Reverse: GCTCTCGCTGTTTCTATGGC |
| *Tollip* | ENSMUSG00000025139 | Forward: TCCGTATAACACCCACACAAC  Reverse: GGTACCAACTGTTCCTCCATAC |
| *Ppp3rl* | ENSMUSG00000033953 | Forward: TCTGGTTCTTTGAGCGTGGA  Reverse: GTTGCCGTCTGTGTCGAATA |
| *Cxcr4* | ENSMUSG00000045382 | Forward: CTGCCCACCATCTACTTCATC  Reverse: CGTCATGCTCCTTAGCTTCTT |
| *Tyrobp* | ENSMUSG00000030579 | Forward: TTCTGGGTGACTTGGTGTTG  Reverse: TGACCCTGAAGCTCCTGATA |
| *GAPDH* | ENSMUSG00000057666 | Forward: GGTGAAGGTCGGTGTGAACG  Reverse: ACCATGTAGTTGAGGTCAATGAAGG |
